# Treatment of a necrotic collection after tangential gastric resection by using an automated endoscopic debridement catheter

**DOI:** 10.1055/a-2550-3809

**Published:** 2025-03-12

**Authors:** Dario Biasutto, Benedetto Neri, Martina Marrelli, Nicolò Citterio, Serena Stigliano, Francesco Maria Di Matteo

**Affiliations:** 1 Therapeutic GI Endoscopy Unit, Fondazione Policlinico Universitario Campus Bio-Medico, Rome, Italy; 2 Department of Systems Medicine, Gastroenterology Unit, University “Tor Vergata” of Rome, Rome, Italy; 3 Operative Endoscopy Department, Fondazione Policlinico Universitario Campus Bio-Medico, Rome, Italy

We report the case of a 59-year-old woman who underwent a proximal subtotal gastrectomy for a gastric stromal tumor. At 15 days after surgery, the patient presented at the emergency room with sepsis, fever, and abdominal pain. Contrast-enhanced computed tomography (CT) scan and subsequent esophagogastroduodenoscopy (EGD) showed a semicircumferential anastomotic dehiscence and a large perigastric collection with super-fluid necrotic material ( Fig. 1 **a, b** ).

**Fig. 1:**
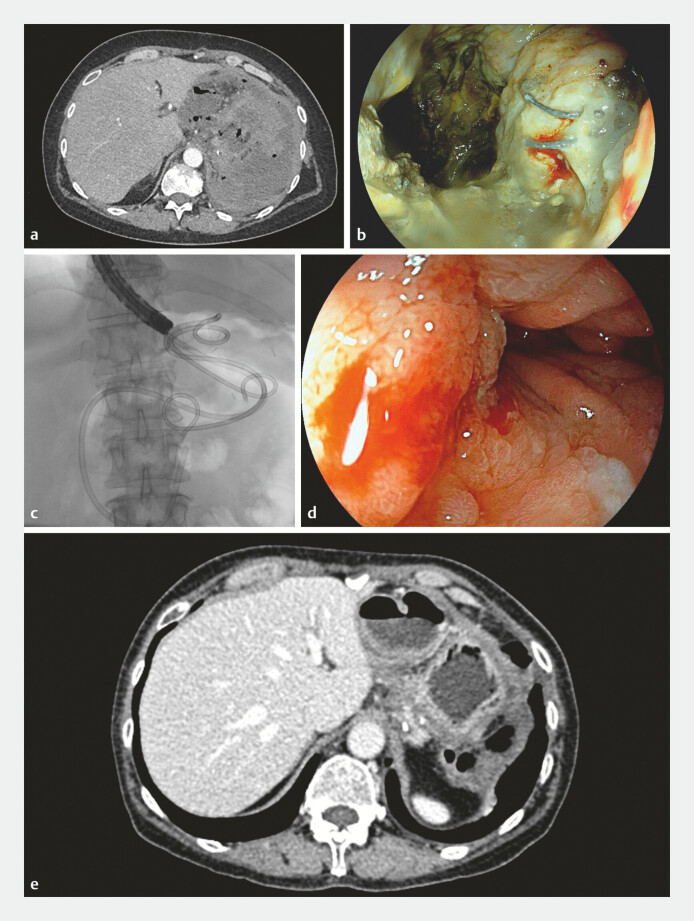
Treatment of a large necrotic collection due to anastomotic leakage after tangential gastric resection. **a** Contrast-enhanced computed tomography (CT) scan showed a large super-fluid collection with gas entrapment. **b** Endoscopic image of the semicircumferential anastomotic leakage after tangential gastric resection, leading to a large, infected collection. **c** Fluoroscopic image of the two double-pigtail stents and the naso-enteral nutrition tube placed after treatment with EndoRotor (Interscope Medical, Inc., Worcester, Massachusetts, USA). **d, e** CT scan and endoscopic images 3 months after treatment, showing complete resolution of the anastomotic leakage and of the infected collection.

To ensure rapid debridement of the purulent collection we used the EndoRotor system ( Video 1 ). The EndoRotor Catheter XT3.1 (Interscope Medical, Inc., Worcester, Massachusetts, USA) is an endoscopic device designed for tissue resection and aspiration, which is achieved through the movement of a motorized rotating and cutting tool driven by an electronically controlled console 1 .

Endoscopic treatment using the EndoRotor system (Interscope Medical, Inc., Worcester, Massachusetts, USA).Video 1

EndoRotor was used through the working channel of a Fujifilm therapeutic gastroscope inserted through the dehiscence for necrosectomy of the perigastric collection ( Video 1 ). Rotation speed was 1750 rpm, suction –700 mmHg, and aspiration volume 50 L/min.

Two necrosectomy sessions were performed on consecutive days until granulating tissue of the collection wall was visible, and signs and symptoms of sepsis improved. Subsequent treatment included positioning of two double-pigtail plastic stents between the cavity and the stomach and a naso-enteral nutrition tube 2 3 ( Fig. 1 **c** ). At 1 month after treatment, the patient tolerated oral nutrition well, and at 3 months, CT scan and EGD showed complete resolution of the collection and leakage ( Fig. 1 **d, e** ).

To the best of our knowledge, this report represents the first case of a postoperative collection successfully treated with the EndoRotor system. Its use in the treatment of pancreatic walled-off necrosis has already been described, suggesting that this device allows safe, fast, and effective removal of necrotic tissue 4 5 .

Anastomotic leakage and collections are usually managed through interventional radiology or surgery, which burden the patient with discomfort and at high risk of complications. We believe that EndoRotor can be a useful tool for ensuring rapid debridement of necrotic tissue while managing postoperative complications in a minimally invasive way.

Endoscopy_UCTN_Code_TTT_1AT_2AF

## Notes

Endoscopy E-Videos https://eref.thieme.de/e-videos
*E-Videos* is an open access online section of the journal *Endoscopy* , reporting on interesting cases and new techniques in gastroenterological endoscopy. All papers include a high-quality video and are published with a Creative Commons CC-BY license. Endoscopy E-Videos qualify for HINARI discounts and waivers and eligibility is automatically checked during the submission process. We grant 100% waivers to articles whose corresponding authors are based in Group A countries and 50% waivers to those who are based in Group B countries as classified by Research4Life (see: https://www.research4life.org/access/eligibility/ ). This section has its own submission website at https://mc.manuscriptcentral.com/e-videos .
